# The Multifarious Functions of Pyruvate Kinase M2 in Oral Cancer Cells

**DOI:** 10.3390/ijms19102907

**Published:** 2018-09-25

**Authors:** Miyako Kurihara-Shimomura, Tomonori Sasahira, Chie Nakashima, Hiroki Kuniyasu, Hiroyuki Shimomura, Tadaaki Kirita

**Affiliations:** 1 Department of Molecular Pathology, Nara Medical University, Kashihara, Nara 634-8521, Japan; miyako@naramed-u.ac.jp (M.K.-S.); c-nakashima@naramed-u.ac.jp (C.N.); cooninh@zb4.so-net.jp (H.K.); 2 Department of Oral and Maxillofacial Surgery, Nara Medical University, Kashihara, Nara 634-8521, Japan; hiroz@naramed-u.ac.jp (H.S.); tkirita@naramed-u.ac.jp (T.K.)

**Keywords:** PKM, Warburg effect, oral cancer

## Abstract

Head and neck cancers, including oral squamous cell carcinoma (OSCC), are the sixth most common malignancies worldwide. OSCC frequently leads to oral dysfunction, which worsens a patient’s quality of life. Moreover, its prognosis remains poor. Unlike normal cells, tumor cells preferentially metabolize glucose by aerobic glycolysis. Pyruvate kinase (PK) catalyzes the final step in glycolysis, and the transition from PKM1 to PKM2 is observed in many cancer cells. However, little is known about PKM expression and function in OSCC. In this study, we investigated the expression of PKM in OSCC specimens and performed a functional analysis of human OSCC cells. We found that the *PKM2*/*PKM1* ratio was higher in OSCC cells than in adjacent normal mucosal cells and in samples obtained from dysplasia patients. Furthermore, PKM2 expression was strongly correlated with OSCC tumor progression on immunohistochemistry. PKM2 expression was higher during cell growth, invasion, and apoptosis in HSC3 cells, which show a high energy flow and whose metabolism depends on aerobic glycolysis and oxidative phosphorylation. PKM2 expression was also associated with the production of reactive oxygen species (ROS) and integration of glutamine into lactate. Our results suggested that PKM2 has a variety of tumor progressive functions in OSCC cells.

## 1. Introduction

Head and neck cancers, including oral squamous cell carcinoma (OSCC), are the sixth most common cancers worldwide [1]. In the United States, approximately 49,670 new patients of OSCC are diagnosed annually, and 9700 die of OSCC each year [2]. In Japan, OSCC accounts for <2% of all cancer morbidities, but this rate is slowly increasing [3]. Despite the advancement in cancer diagnosis and therapy, the overall five-year survival rate for OSCC has remained <50% during the past three decades [4]. Recent advances in molecular biology have revealed the molecular mechanisms underlying the carcinogenesis, tumor progression, and metastasis of OSCC. We previously investigated the several cancer-related molecules to elucidate the molecular mechanism of OSCC development, progression, and metastasis [4,5,6,7,8,9,10]. Furthermore, a recent report has revealed that methylenetetrahydrofolate reductase (MTHFR) gene polymorphisms and methylation of p16 and O^6^-methylguanine-DNA methyltransferase (MGMT) have a pivotal role in OSCC carcinogenesis [11]. However, specific tumor markers of OSCC have not yet been identified. Therefore, clarifying the detailed underlying molecular mechanisms of OSCC is urgently required.

Abnormal energy metabolism of cancer cells is viewed as one of the hallmarks of cancer [12]. Under aerobic conditions, normal cells produce adenosine triphosphate (ATP) from glucose-derived pyruvate by oxidative phosphorylation through the mitochondrial tricarboxylic acid (TCA) cycle [13]. However, in cancer cells, ATP is mainly produced through aerobic glycolysis despite glycolysis yielding lower amounts of ATP from glucose than oxidative phosphorylation [14,15]. This unique phenomenon is called the Warburg effect [16].

Pyruvate kinase (PK) is a glycolytic enzyme that catalyzes the last step in glycolysis: the conversion of pyruvate and ATP from phosphoenolpyruvate and adenosine diphosphate (ADP) [14,17]. Mammals have four PK isoforms, including PKL, PKR, PKM1, and PKM2 [17]. Among these, PKM1 and PKM2 are alternative splicing products of PKM (exons 9 and 10, respectively) [14,17,18]. Generally, the transition from PKM1 to PKM2 is observed in many cancer cells [14,17]. The activation of PKM2 is negatively regulated by CD44 and the increased production of reactive oxygen species (ROS) in colon cancer cells [19,20]. PKM1 is expressed in many normally differentiated tissues and facilitates oxidative phosphorylation [14,17,21], whereas PKM2 is universally expressed in embryonic and immature cells and is necessary for tumor growth in various malignancies [14,17,22,23,24]. In OSCC, PKM2 promotes tumor progression and nodal metastasis, leading to poor prognosis due to enhanced cell growth, invasion, and resistance to apoptosis [25]. However, recent reports have shown that the switch from PKM1 to PKM2 does not occur during tumorigenesis [26], and that PKM2 knockout mice exhibited enhanced tumor growth [27,28]. Other reports have also revealed that the switch of PKM1 to PKM2 in cancers is a tissue-specific event [29,30,31]. Furthermore, PKM1 is associated with tumor progression by the development of resistance to anticancer drugs, enhancement of glucose metabolism, and promotion of autophagy and mitophagy [21,32]. Hence, the role of metabolism and PKM2 in cancer remains controversial.

We therefore hypothesized that the functions of PKM might rely on the metabolic status of cancer cells. The aim of this study was to examine the relationship between PKM1 or PKM2 expression and clinicopathological features in human OSCC specimens. We also assessed the influence of the PKM2 on cancer metabolism in OSCC cells.

## 2. Results

### 2.1. Expression of PKM1 and PKM2 in OSCC

**Figure 1:**
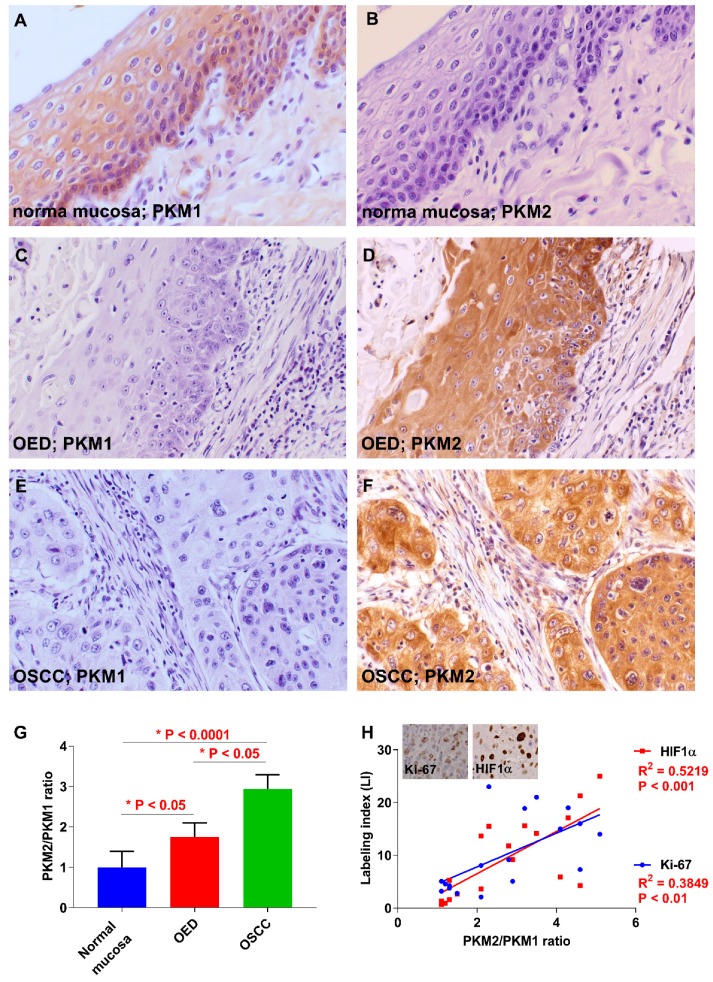
Expression of pyruvate kinase M1 (PKM1) and PKM2 in oral squamous cell carcinoma (OSCC) patients. (**A**) Immunoreactivity to PKM1 is observed in normal oral mucosa adjacent to OSCC. Weak or no expression of PKM1 in oral epithelial dysplasia (OED) (**C**) and OSCC (**E**). (**B**) PKM2 expression was not observed in normal mucosa adjacent to OSCC. Expression of PKM2 was detected in OED (**D**) and OSCC (**F**). (**G**) The PKM2/PKM1 ratio in OSCC cases is lower than in the normal mucosa adjacent to OSCC and in OED. (**H**) The PKM2/PKM1 ratio is closely related to Ki-67 and hypoxia inducible factor 1, the alpha subunit (HIF1α) index in OSCC specimens. Inset shows the expression of Ki-67 and HIF1α. Original magnification is 400×.

First, we examined the expression of PKM1 and PKM2 in several oral diseases (Figure 1A–F). In the normal mucosa, PKM1, but not PKM2, expression was detected in the cytoplasm (Figure 1A,B). PKM1 expression was found in 0% (0/78) and 54.5% (6/11) of cases with OSCC and oral epithelial dysplasia (OED; precancerous lesion), respectively (Figure 1C,D). On the contrary, on immunostaining, PKM2 expression was detected in 82.1% (64/78) and 72.7% (8/11) of patients with OSCC and OED, respectively (Figure 1E,F). Hence, the expression shift from PKM1 to PKM2 was observed at a high frequency in OSCC specimens compared to adjacent normal mucosa and OED. The relationship between PKM2 expression and clinicopathological parameters is summarized in Table 1. PKM2 expression was significantly associated with clinical stage (*P* = 0.0376) and was observed in 93.5% (29/31) of cases with stages III and IV and 74.5% (35/47) with stages I and II (*P* = 0.0376). There were no significant differences between immunoreactivity for PKM2 and other clinicopathological factors in OSCC. A quantitative reverse transcription-polymerase chain reaction (qRT-PCR) using frozen samples confirmed that the PKM2/PKM1 ratio was higher in OSCC than in the adjacent normal mucosal (*P* < 0.0001) and OED cells (*P* < 0.05; Figure 1G). Cell proliferation and anti-hypoxia inducible factor 1 α-subunit (HIF1α) activation promote the expression switching from PKM1 to PKM2 [33,34]; therefore, we examined the markers related to proliferation (Ki-67) and hypoxia (HIF1α). The PKM2/PKM1 ratio was significantly associated with Ki-67 (*P* = 0.0007) and HIF1α *P* = 0.0060) labeling index (LI) in OSCC specimens (Figure 1H). These results suggested that the expression shift from PKM1 to PKM2 occurs at a high frequency in OSCC, and that the switching is promoted by cell proliferation and HIF1α activation.

### 2.2. Tumorigenecity and Proliferative Capacity of OSCC Cells in an Animal Model

**Figure 2:**
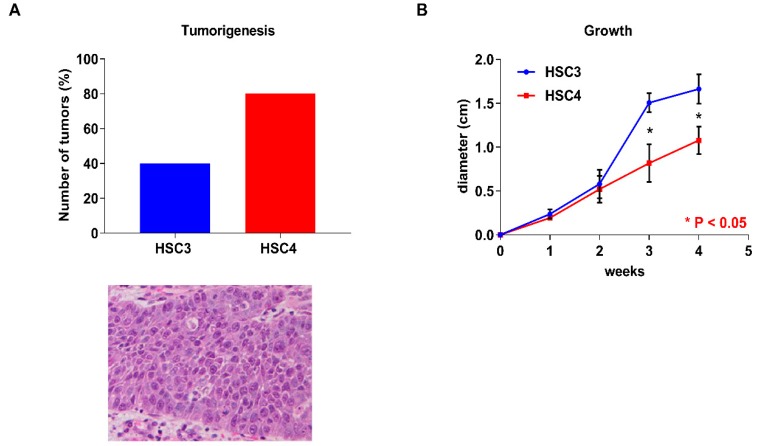
In vivo analysis of HSC3 and HSC4 cells. Tumorigenesis capacity and H&E staining of the transplanted tumor (**A**) and tumor growth (**B**) of HSC3 and HSC4 cells in nude mice. Error bars indicate standard deviations (SDs).

Before in vitro analyses, we investigated the differences in tumorigenesis and the tumor growth abilities of the human OSCC cell line (HSC3 and HSC4 cells) in nude mice. Four tumors were observed in the five mice of the HSC4 cells injected group, whereas two tumors were observed in mice of the HSC3 cells injected group (Figure 2A). These results showed that HSC4 cells had a high tumorigenicity compared to HSC3 cells. In contrast, HSC3 cells grow faster than HSC4 cells (Figure 2B).

### 2.3. Function of PKM2 in OSCC Cells

**Figure 3:**
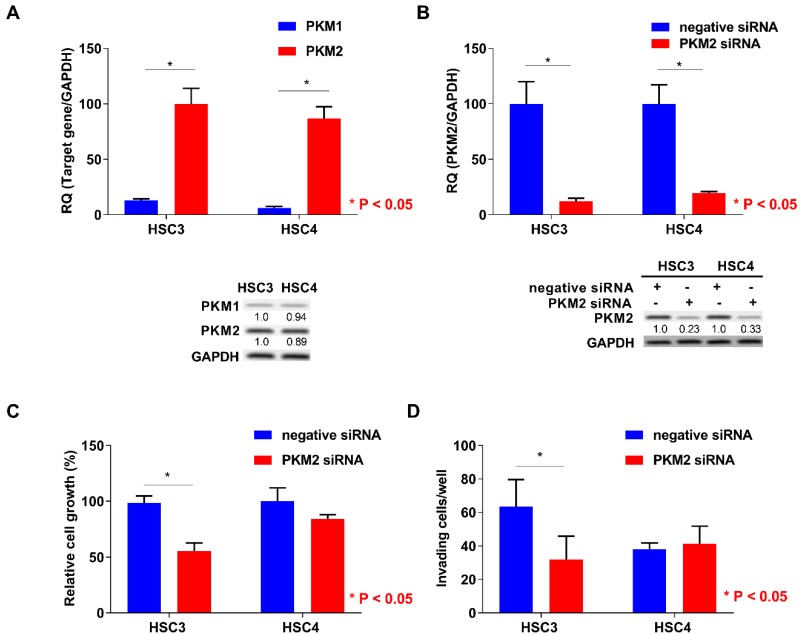

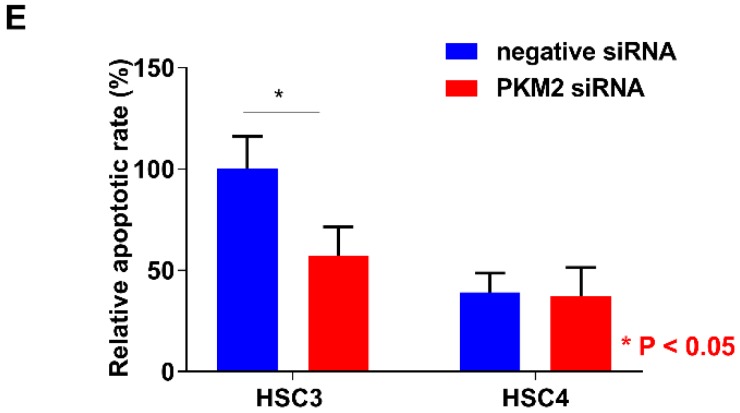
In vitro analysis of pyruvate kinase M2 (PKM2) in HSC3 and HSC4 cells. (**A**) Expression levels of PKM1 and PKM2 in HSC3 and HSC4 cells. GAPDH expression levels represent internal controls. (**B**) Effect of negative or PKM2 siRNA on PKM2 and GAPDH expression in HSC3 and HSC4 cells. Numbers below the panels of immunoblotting are the semiquantified protein expression level. (**C**–**E**) Effects of PKM2 short interfering RNA (siRNA) transfection on cell growth (**C**), invasion (**D**), and apoptosis induction (**E**) in HSC3 and HSC4 cells. Error bars indicate standard deviations (SDs). RQ: relative quantification.

Next, we performed the expression analysis of PKM2 in HSC3 and HSC4 cells. Expression levels of PKM2 were higher than those of PKM1 in both the cells (Figure 3A). To elucidate the functional roles of PKM2, we next performed PKM2 small interfering RNA (siRNA) treatment in OSCC cells. Expression levels of PKM2 were decreased by PKM2 knockdown treatment in HSC3 and HSC4 cells (Figure 3B). Although PKM2 siRNA treatment inhibited cell growth, invasion, and apoptosis-inducing ability in HSC3 cells, PKM2 knockdown had little effect on HSC4 cells (Figure 3C–E).

### 2.4. Relationship between PKM2 Activation and CD44 Expression or ROS Production in OSCC Cells

**Figure 4:**
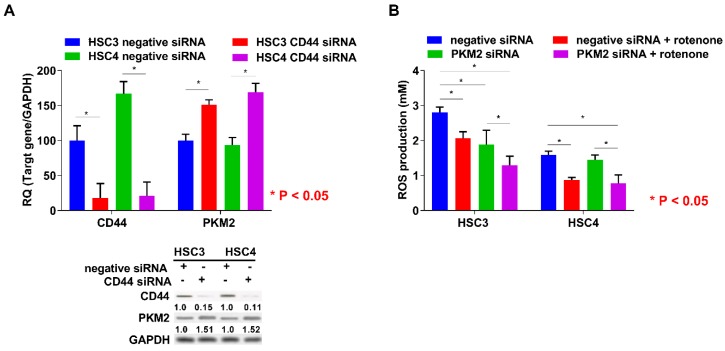
Relationship between PKM2 expression and CD44 knockdown or production of ROS in OSCC cells. (**A**) Effect of negative or CD44 siRNA on CD44, PKM2, and GAPDH expression in HSC3 and HSC4 cells. Numbers below the panels of immunoblotting are the semiquantified protein expression level. (**B**) Effect of PKM2 knockdown on production levels of ROS in HSC3 and HSC4 cells. Error bar: standard deviation. RQ: relative quantification.

Since PKM2 expression is negatively regulated by CD44, which is a marker for cancer stem cells and a repressor of ROS production [19], we next examined the expression regulation of PKM2 by CD44 knockdown treatment. CD44 expression level was higher in HSC4 than in HSC3 cells, and PKM2 expression levels were increased by CD44 knockdown (Figure 4A). Moreover, previous reports have suggested that the activation of PKM2 is increased during the generation of ROS in cancer cells [19,20]. In this study, ROS production was also decreased by PKM2 siRNA treatment, and the inhibition of ROS production was enhanced by rotenone treatment in HSC3 cells, whereas PKM2 knockdown did not affect ROS production in HSC4 cells (Figure 4B). These results suggested that CD44 is associated with expression regaulation of PKM2. Further, PKM2 modulated the production of ROS in OSCC cells.

### 2.5. Energy Metabolism in HSC3 and HSC4 Cells

**Figure 5:**
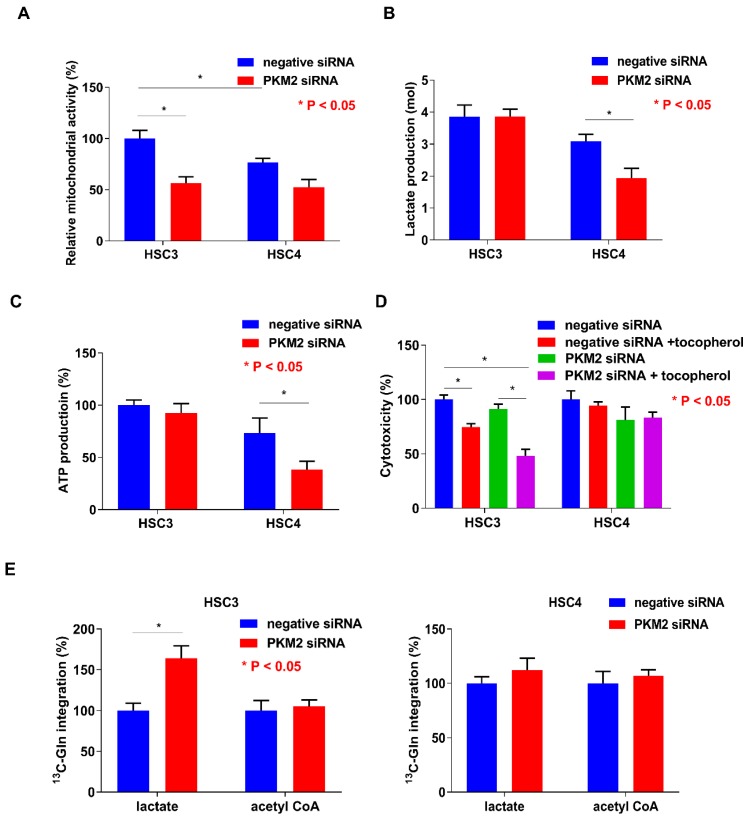
Functions of pyruvate kinase M2 (PKM2) in HSC3 and HSC4 cells. Influence of PKM2 knockdown on mitochondrial activity (**A**), lactate production (**B**), ATP production (**C**), *cytotoxicity* (**D**), and ^13^C-glutamine integration into lactate and acetyl CoA (**E**) in HSC3 and HSC4 cells. Error bar: standard deviation. RQ: relative quantification.

**Figure 6:**
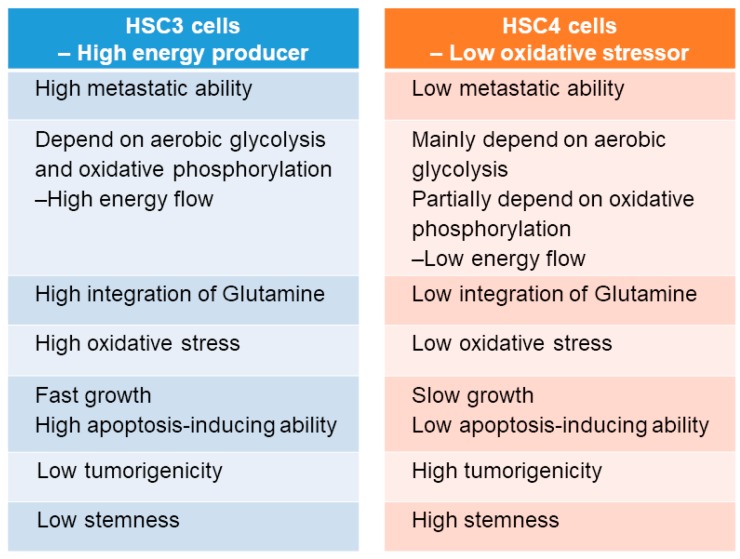
Characteristics of HSC3 and HSC4 cells.

To clarify the differences between HSC3 and HSC4 cells with respect to PKM2 susceptibility, tumorigenicity, and tumor growth, we evaluated the mitochondrial activity and found it to be inhibited by PKM2 knockdown in both the cell lines, without any difference in its activation levels between the two cell lines (Figure 5A). Lactate and ATP production levels in HSC3 cells were higher than those in HSC4 cells, and PKM2 knockdown decreased both these levels in HSC4 cells (Figure 5B,C). Cytotoxicity was reduced when the cells were treated with the anti-oxidant α-tocopherol in PKM2 knockdown HSC3 cells, but not in HSC4 cells (Figure 5D). Cancer cells can utilize glutamine as a carbon source and produce energy through lactate fermentation [35,36]. To investigate whether glutamine can replace glucose to produce pyruvate, we examined the uptake of ^13^C-glutamine in lactate and acetyl CoA in OSCC cells (Figure 5E). While PKM2 siRNA treatment did not influence the incorporation of ^13^C-glutamine into acetyl CoA, it increased ^13^C-glutamine integration into lactate in HSC3 cells. In contrast, treatment with PKM2 siRNA had no effect on ^13^C-glutamine integration into lactate and acetyl CoA in HSC4 cells. The biological characteristics of both the cells are summarized in Figure 6. Based on the above findings, we concluded that PKM2 acts as an oncogene in OSCC, and that different metabolic states of malignancies are associated with tumor-promoting effects of PKM2.

## 3. Discussion

Generally, it is accepted that OSCC arises via multiple genetic and epigenetic alterations, and numerous studies have performed these changes to identify a molecular biomarker of OSCC. Recent reports have indicated that p16, protocadherin FAT1, p53, caspase-8, phosphoinositide 3-kinase (PI3K), Notch1, histone-lysine *N*-methyltransferase 2D (KMT2D), nuclear receptor binding SET domain protein 1 (NSD1), and H-ras are vitally important mutated genes in OSCC patients [37,38]. Another study has shown a higher frequency of methylation at both p16 and MGMT promoter regions in OSCC cases [11]. Moreover, a significant relationship has been found between MTHFR gene polymorphisms and p16. However, no available molecular tumor marker for OSCC has been discovered, and a practical and useful molecular tumor marker is urgently needed.

In this study, we observed a shift in the expression from PKM1 to PKM2 at a high frequency in OSCC cells compared with adjacent normal mucosal and OED cells. PKM2 expression was significantly associated with clinical stage of OSCC. Moreover, we found a significant association between *PKM2* expression and Ki-67 LI or HIF1α activation in OSCC specimens. In OSCC cells, the activation of PKM2 was inhibited by CD44. PKM2 was also promoted in cell growth, invasion, and apoptosis in HSC3 cells, which show a high energy flow and whose metabolism depends on aerobic glycolysis and oxidative phosphorylation. Further, PKM2 regulated the ROS and integration of glutamine into lactate in HSC3 cells. On the basis of these results, we infer that PKM2 participates in the regulation of cancer metabolism in OSCC. However, there were no significant differences in the expression of PKM2 among HSC3 and HSC4 cells. Recent reports have indicated that PKM2 in the cytoplasm also acts as a signaling modulator, as well as a metabolic enzyme, in cancer cells [22,27,39,40]. In addition, PKM2 in the nucleus serves as a transcription regulator to interact with β-catenin, the signal transducer and activator of transcription 3 (STAT3), extracellular signal-regulated kinase1/2 (ERK1/2), Oct4, and nuclear factor-kappa B (NFkB) [40]. The difference of features of HSC3 and HSC4 cells might be due to the kinetics of expression of downstream factors of PKM2 and other Warburg effect-related molecules. Therefore, further studies are warranted. 

Previous reports have clarified that the biological switch from PKM1 to PKM2 is involved in c-myc-mediated cell proliferation and HIF1α activation [33,34], which is consistent with our results. PKM2 also enhances vascular endothelial growth factor (VEGF)-A-dependent tumor angiogenesis by HIF1α activation [40,41]. We previously reported that VEGF-A-mediated angiogenesis is pivotal for tumor progression and nodal metastasis and is associated with poor prognosis in patients with OSCC [5,6,7,8,10]. We also verified that PKM2 positively regulates VEGF-A activation in HSC3 cells (data not shown); thus, the isoform switch of PKM may promote angiogenesis in OSCC.

HSC3 cells have high, whereas HSC4 cells have low, metastatic potential [10]. In the present in vitro study, HSC3 cells performed a high energy-flow metabolism dependent on aerobic glycolysis and oxidative phosphorylation. HSC3 cells also possess high potential for cell growth, invasion, and metastasis, but low potential for apoptosis resistance, tumorigenicity, and stemness. Although HSC4 cells are capable of performing aerobic glycolysis-dependent low energy-flow metabolism and have high tumorigenicity and stemness, their metastatic potential and proliferation ability are low. HSC3 cells can use glutamine as an energy source, whereas HSC4 cells cannot. Interestingly, the inhibition of PKM2 was correlated with a decreased activation of ROS in HSC3 cells under high oxidative stress conditions. Recent reports have suggested that in the absence of CD44, PKM2 is activated, leading to the switch from aerobic glycolysis and oxidative phosphorylation and increased ROS generation [19,20,40], which is mostly consistent with our results. However, PKM2 activation is inhibited by ROS generation in cancer cells [42]. Intracellular accumulation of ROS is generally critical for the proliferation and survival of cancer cells [42], and ROS can suppress cancer metastasis [19,43]. Thus, these dual actions of ROS in malignancies and the association between PKM2 and ROS needs to be further investigated.

The following ten hallmarks are necessary for tumor progression: sustained proliferative signals, evasion of growth suppressors, resistance to cell death, limitless replicative potential, induction of angiogenesis, activation of invasion and metastasis, avoidance of immune destruction, deregulation of cellular energetics, genome instability and mutation, and tumor-promoting inflammation [12]. Nevertheless, the role of metabolic alterations, such as the Warburg effect (aerobic glycolysis), in tumor progression remains unclear. Oxidative phosphorylation in the mitochondria produces up to 36 ATP molecules per glucose molecule, while glycolysis generates only two ATP molecules [44]. However, there is only a little difference in terms of mitochondrial activity, lactate generation, and ATP production in HSC3 and HSC4 cells (Figure 5A–C). Hence, the Warburg effect cannot fully explain cancer promotion and metabolism. We speculate that HSC4 cells mainly perform the aerobic glycolysis dependent metabolism. Additionally, HSC4 cells may partially utilize oxidative phosphorylation. Recently, Lisanti et al. proposed a new model called the “reverse Warburg effect” [45,46]. In their model, cancer cells promoted the Warburg effect in cancer-associated fibroblasts (CAFs) by oxidative stress and autophagy. Increased aerobic glycolysis in CAFs resulted in the increased production of lactate, which was then transferred to adjacent cancer cells and converted into pyruvate [45,46]. They also showed that stromal caveolin-1 (Cav-1) and monocarboxylate transporter 4 (MCT4) are key molecules for the reverse Warburg effect, and their expressions are associated with poor prognosis in patients with triple-negative breast cancer [47]. PKM2 may use either the conventional or the reverse Warburg effect, depending on the cancer environment.

Currently, cisplatin-based therapy combined with other anticancer drugs represents the first-line chemotherapy for OSCC, but the drug resistance of anticancer agents and the antitumor effect in OSCC remain controversial [9]. Recent reports have revealed that PKM2 is associated with cisplatin resistance and gemcitabine tolerance in colon and pancreatic cancer cells, respectively [20,40,48]. Additionally, PKM2 reduces the sensitivity of colon cancer cells to gefitinib via the phosphorylation of STAT3 [40,49]. The inhibition of PKM2 may be useful for improving the resistance to anticancer drugs in patients with OSCC. However, the associations of PKM2 with other anticancer drugs and radiotherapy remain unclear. Hence, reasonable molecular and animal examinations and large-scale clinical studies are warranted.

Although PKM2 is essential for tumor progression in many malignancies [14,17,21,22,23,24], previous reports have indicated that the suppression of PKM2 activity is essential for the efficient growth of cancer cells [22,39]. In addition, the deletion of PKM2 facilitated tumor formation and liver metastasis in a mouse model of breast cancer [27]. Knockout mice of PKM2 also lead to the spontaneous development of liver cancer by remarkable changes in systemic glucose homeostasis, inflammation, and hepatic steatosis [28]. Although PKM1 is expressed in most adult tissues [14,17,21], the conversion of PKM1 into PKM2 is not essential for tumorigenesis [26]. Moreover, PKM1 promotes tumor proliferation, anticancer drug resistance, glucose metabolism, autophagy, and mitophagy in cancer cells [21,32]. We also confirmed that PKM1 is overexpressed in small cell lung carcinoma (data not shown). Thus, the functions and roles of PKM1 and PKM2 in malignancies remain controversial.

In conclusion, PKM2 had a variety of functions in OSCC cells. Moreover, PKM2 expression was associated with tumor progression in human OSCC specimens. However, detailed functions and roles of PKM have not been well-documented. There are many limitations to this study and we must reveal the precise mechanism for why and how PKM2 acts as a tumor progressive factor in OSCC. Additionally, the identification of the role of PKM2/PKM1 in other cancers is essential. Further in vivo and in vitro and large-scale clinical studies will be needed in the future.

## 4. Materials and Methods

### 4.1. Tumor Specimens

Formalin-fixed, paraffin-embedded specimens of OSCC (*n* = 78, 46 males and 32 females, Age range was 43–85 years; mean age was 65.3 years) and OED (*n* = 11, 8 males and 3 females, Age range was 52–73 years; mean age was 54.6 years 6-weii) were randomly selected at Nara Medical University Hospital, Kashihara, Japan. Gene expression analysis was performed on fresh frozen samples of 18 patients with primary OSCC and all samples of OED. None of the patients had undergone radiotherapy and/or chemotherapy before surgical resection. Tumor stage and OSCC histological grade were determined according to the UICC TNM classification system, 7th edition, and WHO criteria, respectively. Medical records were obtained from the patient database managed by the hospital. Written informed consent for using tissue samples was obtained from the patients. All experiments with human samples were performed according to Declaration of Helsinki and approved by the Ethical Committee of Nara Medical University (Approval number 719).

### 4.2. Immunohistochemistry

Consecutive 3-μm sections were cut from each block and subjected to immunohistochemical staining with the EnVision+DualLink system (Dako, Carpinteria, CA, USA). An immunoperoxidase technique was applied following antigen retrieval with microwave treatment (95 °C) in citrate buffer (pH 6.0) for 45 min. Anti-PKM1 (Abgent, San Diego, CA, USA), anti-PKM2 (Cell Signaling Technology, Danvers, MA, USA), HIF1α (Thermo Fischer Scientific, Waltham, MA, USA), and anti-Ki-67 antibody (DAKO) diluted at 0.5 μg/mL were used as primary antibodies. After 2 h of incubation at room temperature, the sections were incubated with a secondary antibody for 30 min. The specimens were color-developed with diaminobenzidine (DAB) solution (Dako) and counterstained with Meyer’s hematoxylin (Sakura Finetek, Tokyo, Japan). Immunostaining of all samples was performed using the same conditions of the antibody reaction and DAB exposure. Immunoreactivities of PKM1 and PKM2 were classified according to the Allred’s score (AS) [50]: grade 1 for AS = 0, 2 for AS = 2–4, and 3 for AS = 5–8. Grade 2 and 3 cases were regarded as immunopositive [5]. We also observed 20 microscopic fields per case at 200× magnification and counted the tumor cells per case; the results were expressed as the percentage of tumor cells, with positive nuclei defined by Ki-67 and HIF1α LI [10].

### 4.3. Cell Culture and Reagents

HSC3, which is established from metastatic focus and possesses metastatic potential, and HSC4, which is established from a primary site and low metastatic potential, cells were human tongue cancer cell lines derived from the same patient [7,10]. Further, both cells have different lactate fermentation, redox, and stemness statuses [15]. Hence, HSC3 and HSC4 cells were used in the present study. Both cells were obtained from the Health Science Research Resources Bank and maintained in Dulbbeco’s modified Eagle’s medium (DMEM; Wako Pure Chemical, Osaka, Japan) supplemented with 10% fetal bovine serum (FBS; Nichirei Biosciences, Tokyo, Japan) and penicillin/streptomycin (Wako Pure Chemical) under 5% CO_2_ and 95% air at 37 °C. Cells were treated with rotenone (50 nM; R&D Systems, Minneapolis, MN, USA) and α-tocopherol (50 μM; Sigma-Aldrich, Darmstadt, Germany) to observe ROS production and cytotoxicity, respectively. Furthermore, both cell lines were cultured in a regular medium supplemented with 2 mmol/L ^13^C-glutamine (Isotech, Southport, Merseyside, UK) for 12 h, and lactate and acetyl CoA concentrations were measured [36].

### 4.4. siRNA

Stealth Select RNAi (siRNA) for PKM1 and PKM2 was purchased from Invitrogen (Carlsbad, CA, USA) and AllStars Negative Control siRNA from Qiagen (Valencia, CA, USA). HSC3 and HSC4 cells were cultured in six-well plates until 40% confluence. Transfection of 10-nM siRNA was performed with Lipofectamine 3000 (Invitrogen) according to the manufacturer’s recommendations. Briefly, the mixture of Opti-MEM Reduced Serum Medium (Invitrogen) including siRNA and Lipofectamine 3000 was added into the wells. After 24 h from transfection, the cells were used for further analyses.

### 4.5. Cell Growth and Apoptosis Assays

Cells were seeded onto 96-well tissue culture plates at a density of 2000 cells per well and incubated for 48 h at 37 °C. Cell growth was assessed using the Cell Counting Kit-8 (Dojindo, Kumamoto, Japan), and apoptotic cells were detected using the APOPercentage Apoptosis Assay (Biocolor, Carrickfergus, County Antrim, UK). Absorbance at 450 nm (for cell growth quantification) and 550 nm (for apoptosis quantification) was recorded for each well using a Multiskan GO Microplate Spectrophotometer (Thermo Fischer Scientific). All experiments were performed in triplicate.

### 4.6. Cell Invasion Assay

A modified Boyden chamber assay was performed using BD BioCoat Cell Culture Inserts coated with Type IV Collagen (BD Biosciences, Bedford, MA, USA). Briefly, cells were suspended in 500 μL of DMEM and placed in the insert. After incubation for 48 h at 37 °C, the filters were dyed with a Diff-Quick Staining Kit (Siemens Healthcare Diagnostics, Newark, DE, USA). The stained cells were counted in whole inserts at 100× magnification. Each experiment was repeated at least three times.

### 4.7. qRT-PCR

Total RNA was extracted using the RNeasy Mini Kit (Qiagen, Valencia, CA, USA) and total RNA (1 μg) was synthesized with the ReverTra Ace qRT Kit (Toyobo, Osaka, Japan). A quantitative reverse transcription-polymerase chain reaction (qRT-PCR) was performed on StepOne Plus Real-Time PCR Systems (Applied Biosystems, Foster City, CA, USA) using TaqMan Fast Universal PCR Master Mix (Applied Biosystems) and analyzed via the relative standard curve quantification method. PCR conditions were set according to the manufacturer’s instructions and the *glyceraldehyde-3-phosphate dehydrogenase* (*GAPDH*) mRNA level was amplified for the internal control. TaqMan Gene Expression Assays of *PKM1*, *PKM2*, *CD44*, and *GAPDH* were purchased from Applied Biosystems. All PCR experiments were done in triplicate.

### 4.8. Immunoblotting

Whole-cell lysates were obtained using the M-PER Mammalian Protein Extraction Reagent (Thermo Fisher Scientific Inc., Rockford, IL, USA). Each lysate (50 μg) was subjected to 12.5% SDS-PAGE and immunoblotting by electrotransfer to polyvinylidene fluoride (PVDF) membranes (Thermo Fisher Scientific). The filters were incubated with anti-PKM1 (Abgent) and anti-PKM2 antibodies (Cell Signaling Technology) further amplified with peroxidase-conjugated IgG (MBL, Nagoya, Japan). The immune complex was visualized by the ECL Western blotting detection system (GE Healthcare, Amersham place, UK). An anti-GAPDH antibody (Santa Cruz Biotechnology Santa Cruz, USA) was used as an internal control. The specific bands on the immunoblotted membrane detected by each antibody were quantified with NIH image computer software (National Institutes of Health, Bethesda, MD). The signal intensities were compensated for by GAPDH intensities as internal controls.

### 4.9. Enzyme-Linked Immunosorbent Assay (ELISA) and Measurement of ATP and ROS Production

ELISA for lactate (Biovision, Milpitas, CA, USA) and acetyl CoA (Abnova, Taipei, Taiwan) was performed according to the manufacturer’s protocol. Proteins were extracted using M-PER Mammalian Protein Extraction Reagent (Thermo Fisher Scientific). The Cytochrome C ELISA Kit (R&D Systems) was used to quantify the mitochondrial activity. The production of ATP and ROS was determined using the ATP Assay Kit (Abnova) and Lipid Peroxidation (MDA) Assay Kit (Biovision), respectively.

### 4.10. Animal Models

Five-week-old male BALB/cSlc-nu/nu mice were purchased from Japan SLC (Shizuoka, Japan) and maintained in accordance with the institutional guidelines approved by the Committee for Animal Experimentation of Nara Medical University and current regulations and standards established by the Ministry of Health, Labor, and Welfare of Japan. Each experimental group comprised five mice. To prepare the tumor model, HSC3 and HSC4 cells (1 × 10^6^ cells) suspended in Hank’s balanced salt solution (Wako Pure Chemical) were inoculated into the scapular subcutaneous tissue of the mice. At week 4, the tumors were excised for assessment. All tumors were fixed in formalin and embedded in paraffin for H&E staining for the evaluation of tumorigenesis.

### 4.11. Statistical Analysis

Statistical analysis was performed with the JMP13 software (SAS Institute, Cary, NC, USA). Statistical differences were evaluated using the Fisher’s exact test, Student’s *t*-test, Welch’s *t*-test, one-factor ANOVA test, and Mann–Whitney *U* test. *P*-values of <0.05 were regarded as statistically significant.

## Figures and Tables

**Table 1:** Relationship between PKM2 expression and clinicopathological parameters.

Parameters	PKMN2 Expression		
	Negative	Positive	*P* Value **
Gender			
Male	6	40	
Female	8	24	0.2331
Age			
≤65	3	28	
>65	11	36	0.1442
Site			
Tongue	4	27	
Other	10	37	0.3861
Histological differentiation *			
Well, Moderately	11	57	
Poorly	3	7	0.3735
T classification			
T1–T2	12	37	
T3–T4	2	27	0.0682
Clinical stage			
I–II	12	35	
III–IV	2	29	0.0376
Nodal metastasis			
Negative	12	50	
Positive	2	14	0.7220

Relationship between the expression of PKM2 and parameters was calculated by Fisher’s exact test. T classification and clinical stage were classified according to the TNM classification. * Histological differentiation: Well, well-differentiated squamous cell carcinoma; Moderately, moderately differentiated squamous cell carcinoma; Poorly, poorly differentiated squamous cell carcinoma. ** *P* value < 0.05 was regarded as statistically significant.

## Abbreviations

AS	Allred’s score
ADP	adenosine diphosphate
ATP	adenosine triphosphate
CAFs	cancer-associated fibroblasts
DAB	diaminobenzidine
DMEM	Dulbbeco’s modified Eagle’s medium
ELISA	Enzyme-linked immunosorbent assay
ERK1/2	extracellular signal-regulated kinase1/2
FBS	fetal bovine serum
GAPDH	glyceraldehyde-3-phosphate dehydrogenase
HIF1α	hypoxia inducible factor 1 α-subunit
KMT2D	histone-lysine N-methyltransferase 2D
MGMT	O^6^-methylguanine-DNA methyltransferase
MTHFR	methylenetetrahydrofolate reductase
NFkB	nuclear factor-kappa B
OED	oral epithelial dysplasia
OSCC	oral squamous cell carcinoma
PI3K	phosphoinositide 3-kinase
PK	pyruvate kinase
PVDF	polyvinylidene fluoride
qRT-PCR	Quantitative reverse transcription-polymerase chain reaction
ROS	reactive oxygen species
STAT3	signal transducer and activator of transcription 3
TCA	tricarboxylic acid
VEGF	vascular endothelial growth factor
